# Microstructure and Solute Segregation around the Melt-Pool Boundary of Orientation-Controlled 316L Austenitic Stainless Steel Produced by Laser Powder Bed Fusion

**DOI:** 10.3390/ma16010218

**Published:** 2022-12-26

**Authors:** Kazuhisa Sato, Shunya Takagi, Satoshi Ichikawa, Takuya Ishimoto, Takayoshi Nakano

**Affiliations:** 1 Research Center for Ultra-High Voltage Electron Microscopy, Osaka University, 7-1 Mihogaoka, Ibaraki 567-0047, Japan; 2 Division of Materials and Manufacturing Science, Graduate School of Engineering, Osaka University, 2-1 Yamadaoka, Suita 565-0871, Japan; 3 Anisotropic Design & Additive Manufacturing Research Center, Osaka University, 2-1 Yamadaoka, Suita 565-0871, Japan; 4 Aluminium Research Center, University of Toyama, 3190 Gofuku, Toyama 930-8555, Japan

**Keywords:** additive manufacturing, laser powder-bed fusion, cellular microstructure, solidification segregation, transmission electron microscopy

## Abstract

For this article, we studied the microstructure and solute segregation seen around the melt pool boundary of orientation-controlled 316L austenitic stainless steel produced by laser powder bed fusion, using transmission electron microscopy and energy-dispersive x-ray spectroscopy. We found that the solidification cellular microstructures could be visualized with the aid of solute segregation (Cr and Mo) during solidification. Mn–Si–O inclusions (10–15 nm in diameter) were distributed along the lamellar boundaries, as well as in the dislocation cell walls. It is believed that the grain growth of the inclusions can be effectively suppressed by rapid quenching during the laser powder-bed fusion process. A thin region without cellular microstructures was observed at the melt-pool boundary. The cellular spacing widened near the bottom of the melt-pool boundary, owing to the decrease in the cooling rate. Atomic-structure analysis at the lamellar boundary by high-resolution transmission electron microscopy revealed a local interfacial structure, which is complementary to the results of electron back-scatter diffraction.

## 1. Introduction

Laser powder bed fusion (LPBF), which is a typical metal-additive manufacturing (AM) technique using a laser as the heat source, is a promising materials-manufacturing process characterized by flexible shaping ability and high processing speed [1,2,3]. LPBF is characterized by a unique solidification process that achieves both a high temperature gradient (~10^7^ K/m) and a high solidification rate (~10^−1^ m/s). Therefore, research on LPBF covers a wide range of topics, such as fluid dynamics, heat transfer, material microstructures, and mechanical properties. The melt flow and formation mechanism of the pores, spatters, and denudation zones during the LPBF process have been studied to better understand the process and control the microstructure [4,5]. Microstructure design is also a key issue in terms of controlling mechanical properties, such as the cellular structure strengthening mechanism reported by Voisin et al. [6]. For crystalline materials, control of the crystal orientation, as well as the morphology, is essential for improving their properties. Characteristic hierarchical structures and the segregation of solute elements have been reported for 316L austenitic stainless steel (SS), produced by the LPBF method [7,8,9]. Recently, a unique crystallographic lamellar microstructure (CLM) has been reported for 316L SS by controlling the scanning strategy of LPBF [10,11]. The material has a wide range of potential applications in the chemical, petrochemical, marine, and medical industries because of its corrosion resistance and mechanical properties; hence, 316L has been actively studied in the field of AM [6,7,8,9,10,11,12,13]. The CLM of LPBF 316L SS is characterized by its crystallographic texture, with <011> and <100> orientations in the build direction (crystal-growth direction) and laser-scanning direction, respectively [10,11]. The overall feature of the microstructure has been characterized using scanning electron microscopy combined with electron back-scatter diffraction (SEM-EBSD). However, the details of the local interfacial structure, including the solute segregation of the CLM, are still unclear. The latest electron-microscopy techniques enable the local characterization of structural and chemical irregularities [14,15]. The purpose of this study is to clarify the microstructure and solute segregation of orientation-controlled 316L SS, produced by controlling the scanning strategy of LPBF (hereafter, LPBF 316L SS) by transmission electron microscopy (TEM) and energy-dispersive x-ray spectroscopy (EDS). Here, we show that the solidification cellular microstructures can be visualized with the aid of solute segregation, even in the case of orientation-controlled microstructures. We also discuss the cellular spacing of the CLM, based on the cooling rate.

## 2. Materials and Methods

LPBF 316L SS was fabricated using an EOS M290 printer (EOS GmbH, Krailling, Germany) by scanning the laser beam bidirectionally along the *X*-axis (*X*-scan strategy). The build direction was defined as the *Z*-axis; hence, a parabolic melt-pool boundary can be observed on the *YZ* plane [10,11]. The nominal composition of the powder was 18Cr–14Ni-2.5Mo-0.03C (wt %). The details of the LPBF process have been described in a previous paper [11]. A crystal grown under low energy density (conduction mode) conditions, characterized by a flat melt-pool bottom, was used for the subsequent TEM observations. Plan-view TEM specimens were prepared from the *YZ* plane using a focused-ion beam (FIB) instrument (Scios2 Dual Beam, Thermo Fisher Scientific, Hillsboro, OR, USA) with Ga ions. Thinning was first performed with an energy of 30 keV and finished with an energy of 2 keV. Chemical etching (21% HF, 29% HNO_3_, 50% distilled water) was performed to reveal the solidification cellular structures before FIB micro-sampling. The structure and morphology of the prepared specimens were characterized using a JEM ARM200F transmission electron microscope (JEOL Ltd., Tokyo, Japan) operating at 200 kV. The TEM images were acquired with a 4k CMOS camera (OneView, Gatan, Inc., Pleasanton, CA, USA). For scanning TEM (STEM) imaging, we set the beam convergence to a semi-angle of 23 mrad. Bright-field (BF) and high-angle annular dark-field (HAADF) STEM images were acquired, with detector semi-angles of 0–17 and 68–170 mrad, respectively. Compositional analysis was performed with an energy-dispersive X-ray spectrometer (JEOL JED-2300) attached to the electron microscope. The chemical composition was evaluated, based on thin-film approximation [16].

## 3. Results and Discussion

**Figure 1:**
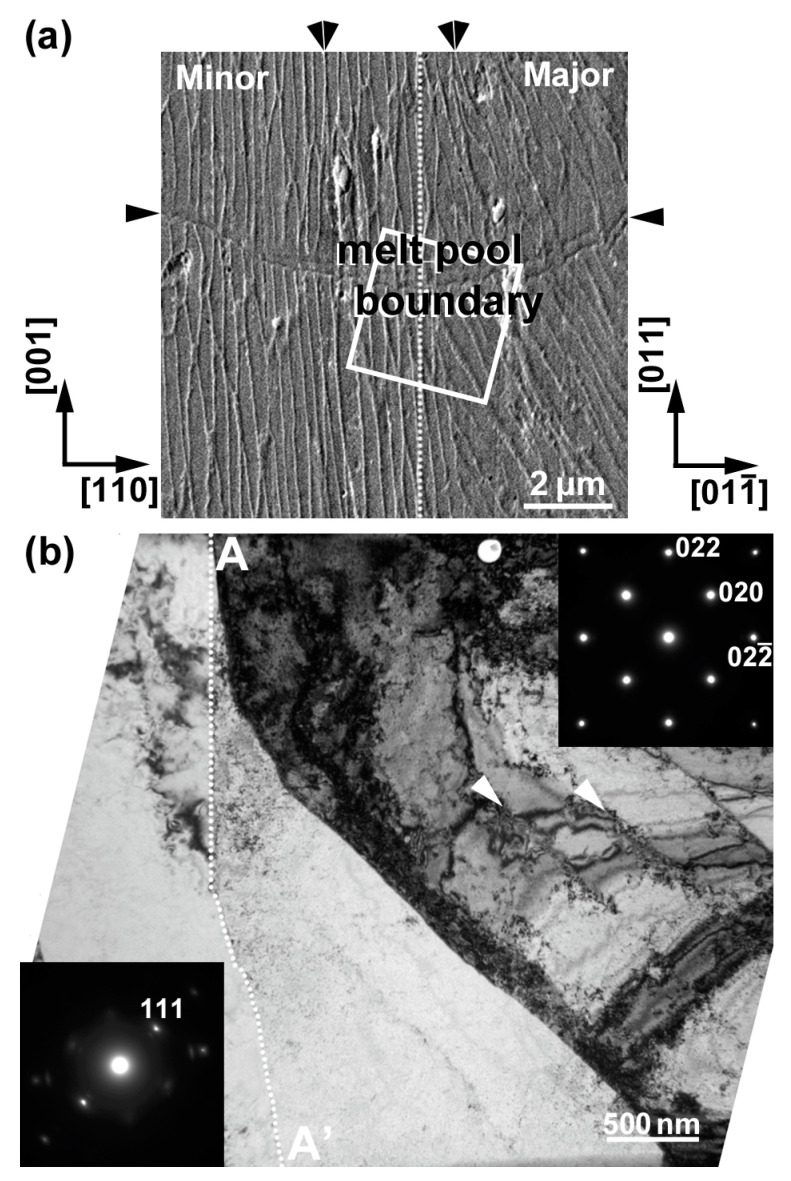
(**a**) Secondary electron (SE) image of the region containing the melt-pool boundary (*YZ* plane). (**b**) BF-TEM image and the corresponding SAED patterns. The vertical line A-Aʹ separates the minor layer (left) and the major layer (right). The double arrowheads indicate the representative solidification cellular microstructures.

A secondary electron (SE) image around the bottom of the melt-pool boundary (*YZ* plane) is shown in Figure 1a. The solidification cellular microstructures were observed as numerous vertical and inclined lines with bright contrast (representative solidification cellular microstructures are indicated by double arrowheads). The crystallographic orientations are indicated in the figure. The vertical dotted line shows the lamellar boundary between a minor layer ([001] orientation in the build direction, left side of the boundary) and a major layer ([011] orientation in the build direction, right side of the boundary). The terms “minor layer” and “major layer” have been used in previous studies [10,11]. The melt-pool boundary is indicated by arrowheads. A plan-view TEM specimen was fabricated from the rectangular area containing both the melt-pool boundary and lamellar boundary (the square indicates the approximate location of the area for FIB micro-sampling). A BF-TEM image of the specimen, prepared by FIB, is shown in Figure 1b. The boundary indicated by the dotted line (A-Aʹ) is the lamellar boundary between the minor (left side) and major (right side) layers. The observation direction of the major layer was in the [100] direction. The arrowheads indicate the dislocation cell walls inside the major layer. The crystal orientation greatly changed at the minor layer, as shown in the SAED pattern (bottom left insert). The (111) atomic plane appeared at the interface, which differed from that derived by SEM-EBSD analysis [10,11]: <011>_major_ // <001>_minor_ in the build direction has been reported for the CLM [10]. This result suggests that high-resolution TEM (HRTEM) observation can characterize local interfacial structures, which will be shown later. It should be mentioned that the melt-pool boundary would exist in this field of view, in comparison with the SEM image observed before FIB micro-sampling, although it is not visible in the BF-TEM image. Epitaxial growth of the lamellar microstructure across the melt-pool boundary caused no obvious diffraction contrast or strain contrast.

**Figure 2:**
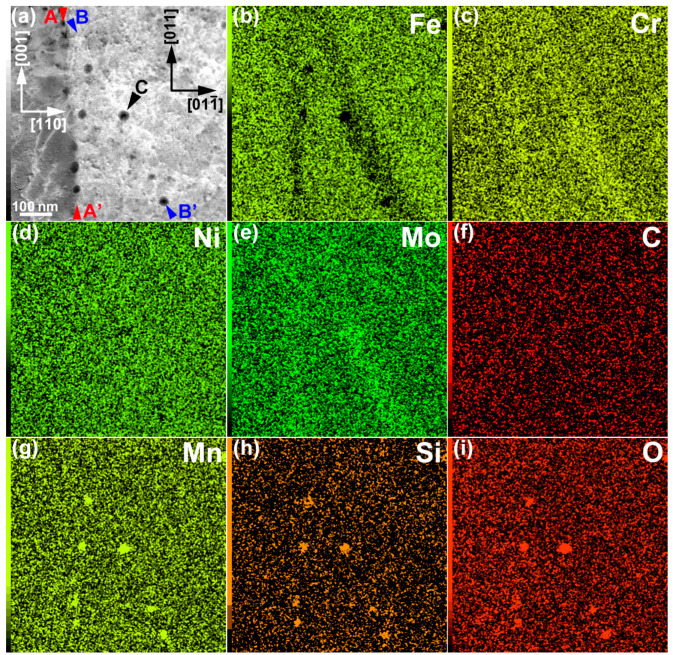
STEM-EDS elemental maps: (**a**) HAADF-STEM image, (**b**) Fe map, (**c**) Cr map, (**d**) Ni map, (**e**) Mo map, (**f**) C map, (**g**) Mn map, (**h**) Si map, and (**i**) O map. The elemental maps are shown in wt %.

The results of the STEM-EDS elemental mapping of an area, including a lamellar boundary and a dislocation cell wall, are shown in Figure 2. The elemental maps are shown as a weight percentage (wt %). The boundary along the line connecting the arrowheads A and Aʹ (A–Aʹ) is the lamellar boundary between the minor layer (on the left side of the boundary) and the major layer (on the right side of the boundary). Conversely, the boundary along the line connecting the arrowheads B and Bʹ (B–Bʹ) is the dislocation cell wall inside the major layer. Cr and Mo were enriched along the lamellar boundary, as well as in the dislocation cell wall. The segregation of Cr and Mo was ~1 wt % and 1–2 wt %, respectively. The segregation tendency was qualitatively reproduced by a modified Scheil–Gulliver calculation, assuming a cooling rate of 10^6^ K/s and a cell distance of 300 nm [17]. Similar results have been reported by Depinoy et al. [18]. Thus, the segregation of Cr and Mo occurred at the cell boundaries, similar to the findings in recent reports on LPBF 316L SS without the CLM [7,8]. Another feature of the mapping results is the existence of dark-contrast particulate distributed along the lamellar boundary and in the dislocation cell wall in the HAADF-STEM image (representative dark-contrast particulate is indicated by arrowhead C). The atomic-number contrast (*Z* contrast) of the HAADF-STEM image suggests that these dark-contrast regions were composed of elements lighter than Fe. In fact, the elemental maps showed that Mn, Si, and O were enriched in these dark-contrast regions. These regions could be nanoscale MnO–SiO_2_-type inclusions (rhodonite), as reported in previous studies on LPBF 316L SS [8,19]. The existence of MnS inclusions can be excluded because S was not explicitly detected via EDS analysis. It was found that the scanning strategy did not significantly change solute segregation and the distribution of nanoscale inclusions.

**Figure 3:**
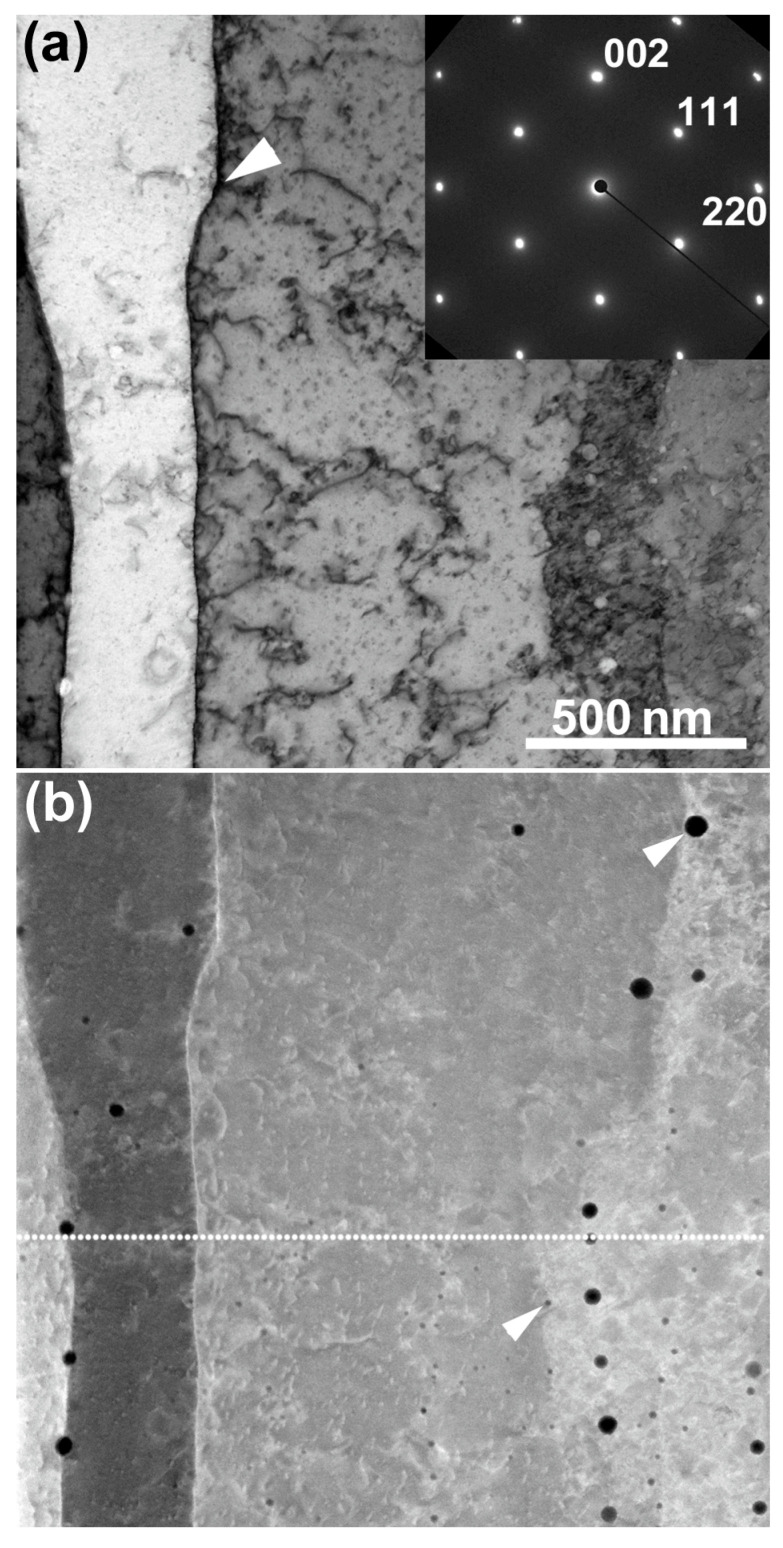
(**a**) The BF-STEM image and the corresponding SAED pattern, obtained from an area including the melt-pool boundary. The beam incidence was in the $\left[1\overset{-}{1}0\right]$ direction. (**b**) HAADF-STEM image of the area shown in Figure 3a. The dotted line indicates the approximate location of the melt-pool boundary. The arrowheads indicate particulate Mn–Si–O inclusions.

A BF-STEM image and the corresponding SAED pattern obtained from a minor layer, including the melt-pool boundary, are shown in Figure 3a. The [001] direction corresponds to the build direction. Cellular structures had formed, while the crystal orientation was the same across the dislocation cell walls. Dislocations were observed in the cells, as well as at the cell boundaries. A noticeable feature was that the cell boundary was bent at the position of the arrowhead, and hence the cellular spacing changed. It is presumed that this bending is related to the position of the melt-pool boundary. However, the location of the melt-pool boundary was not visible in this image. A HAADF-STEM image, simultaneously obtained with the BF-STEM image depicted in Figure 3a, is shown in Figure 3b. The approximate location of the melt-pool boundary is indicated by the dotted line, based on the SE image observed before FIB micro-sampling. The melt-pool boundary was located just below the curved part of the cell boundary. However, the existence of the melt-pool boundary was not obvious, and its presence was difficult to determine from the *Z*-contrast of the HAADF-STEM image. Based on the TEM and STEM results, the melt-pool boundary could not be observed by either the diffraction contrast, strain contrast, or *Z*-contrast. The distribution of the Mn–Si–O inclusions can be observed as a dark contrast along the cell boundaries (the typical large and small inclusions are indicated by arrowheads). Inclusions were easier to recognize in the HAADF-STEM image than in the BF-STEM image, owing to incoherent imaging without diffraction contrast.

**Figure 4:**
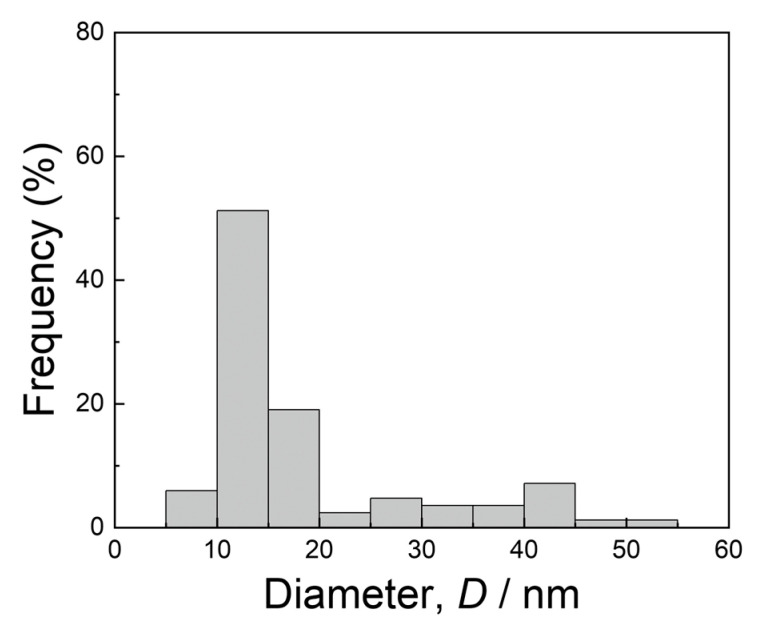
Histogram of the diameters of the Mn–Si–O inclusions, measured from the HAADF-STEM images.

A histogram of the diameters of the Mn–Si–O inclusions, measured from the HAADF-STEM images, is shown in Figure 4. The inclusion diameters were distributed in the range from 10 to 50 nm, with a peak at 10–15 nm. These diameters were extremely small compared with those observed in conventional stainless steel [20]; hence, such nanometer-sized inclusions should not contribute to pitting corrosion. This was verified in a recent study by Tsutsumi et al. [13], based on the anodic polarization test. It is believed that the grain growth of inclusions can be suppressed effectively by rapid quenching during the LPBF process. This is one of the benefits of AM, offering both a very high temperature gradient and a high solidification rate.

**Figure 5:**
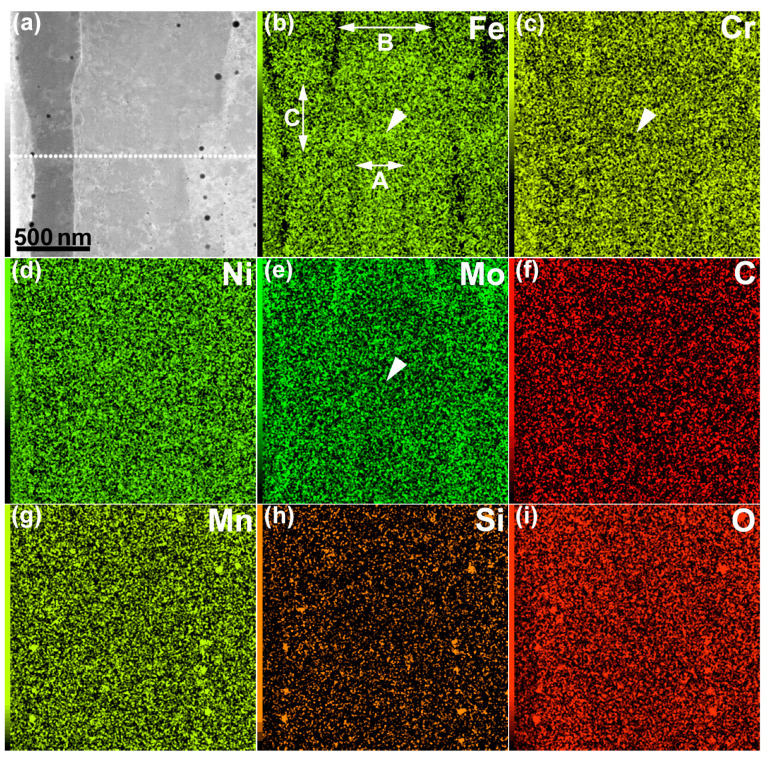
STEM-EDS elemental maps: (**a**) HAADF-STEM image, (**b**) Fe map, (**c**) Cr map, (**d**) Ni map, (**e**) Mo map, (**f**) C map, (**g**) Mn map, (**h**) Si map, and (**i**) O map. The elemental maps are shown in wt %.

The results of the STEM-EDS elemental mapping of an area, including the melt-pool boundary, are shown in Figure 5. The elemental maps are shown in wt %. The field of view was the same as that shown in Figure 3b. The dotted line of the HAADF-STEM image indicates the approximate location of the melt-pool boundary. The Cr and Mo contents slightly decreased by ~0.5 wt % at the melt-pool boundary, while they increased by 1–2 wt % at the cell boundaries. A similar tendency was reported by Godec et al. [8]. This can be explained by segregation: solidification starts at the bottom of the melt pool and cellular solidification proceeds. This is in good agreement with the simulation results [17,18]. The increase in the Fe content (~1 wt %) at the melt-pool boundary was visible on the Fe map, as indicated by the arrowhead in Figure 5b, while the decreases in the Cr and Mo contents (~0.5 wt %) were faint on the elemental maps (Figure 5c,e). The decreases in the Cr and Mo concentrations lead to deterioration in the corrosion resistance, making the material more susceptible to corrosion by acid. Conversely, increases in the Cr and Mo concentrations improve the corrosion resistance. In fact, the cellular boundary was observed with bright contrast in the SE image after chemical etching, indicating that it was a convex part. By contrast, the melt-pool boundary was observed with dark contrast, indicating that it was a concave part. These features are clearly visible in the SE image shown in Figure 1a. The fact that the melt-pool boundary and cellular boundary became observable owing to corrosion suggests that three-dimensional (3D) observation of the solidification cellular microstructures by FIB-SEM serial sectioning may be difficult. Therefore, the development of a novel technique for the 3D structural analysis of LPBF-produced materials, including the tomographic methodology [21], is urgently needed.

Another feature of the Fe map was the changes in the cellular spacing (*λ*) above and below the melt-pool boundary. The spacing of the cell below the melt-pool boundary (indicated by double-headed arrow A in Figure 5b) was 330 nm, while that of the cell above the melt-pool boundary (indicated by double-headed arrow B in Figure 5b) was 680 nm. For LPBF 316L SS, Tsutsumi et al. [13] discussed the relationship between the cooling rate ($\dot{T}$) and the cellular spacing, based on the equation $\lambda =80{\dot{T}}^{-0.33}$ [22]. Using this equation, the cooling rate of the cell just below the melt-pool boundary was calculated to be 1.7 × 10^7^ K/s, and that above the boundary was calculated to be 1.9 × 10^6^ K/s. The mobility of the solid/liquid interface must be low near the bottom of the melt pool; hence, the widening of the cellular spacing can be attributed to the decrease in the cooling rate. The low mobility of the solid/liquid interface will stabilize planar solidification. In fact, the transition from planar to cellular solidification at the bottom of the melt pool has been reported by Vrancken et al. [23]. In Figure 5b, there is a region with almost no cellular contrast above the melt-pool boundary, as indicated by double-headed arrow C, which is possibly a planar solidification zone, as mentioned above. The width of this region was 470 nm, which was close to the range (0.5–1 μm) reported in the literature [23]. In the SE image shown in Figure 1a, two boundary contrasts are observed at the bottom of the melt pool, and almost no cellular boundary is observed between them. This is consistent with the elemental map shown in Figure 5b; hence, it is presumed that a transition from planar to cellular solidification occurred. To verify this point, further investigation, including simulation, is necessary.

**Figure 6:**
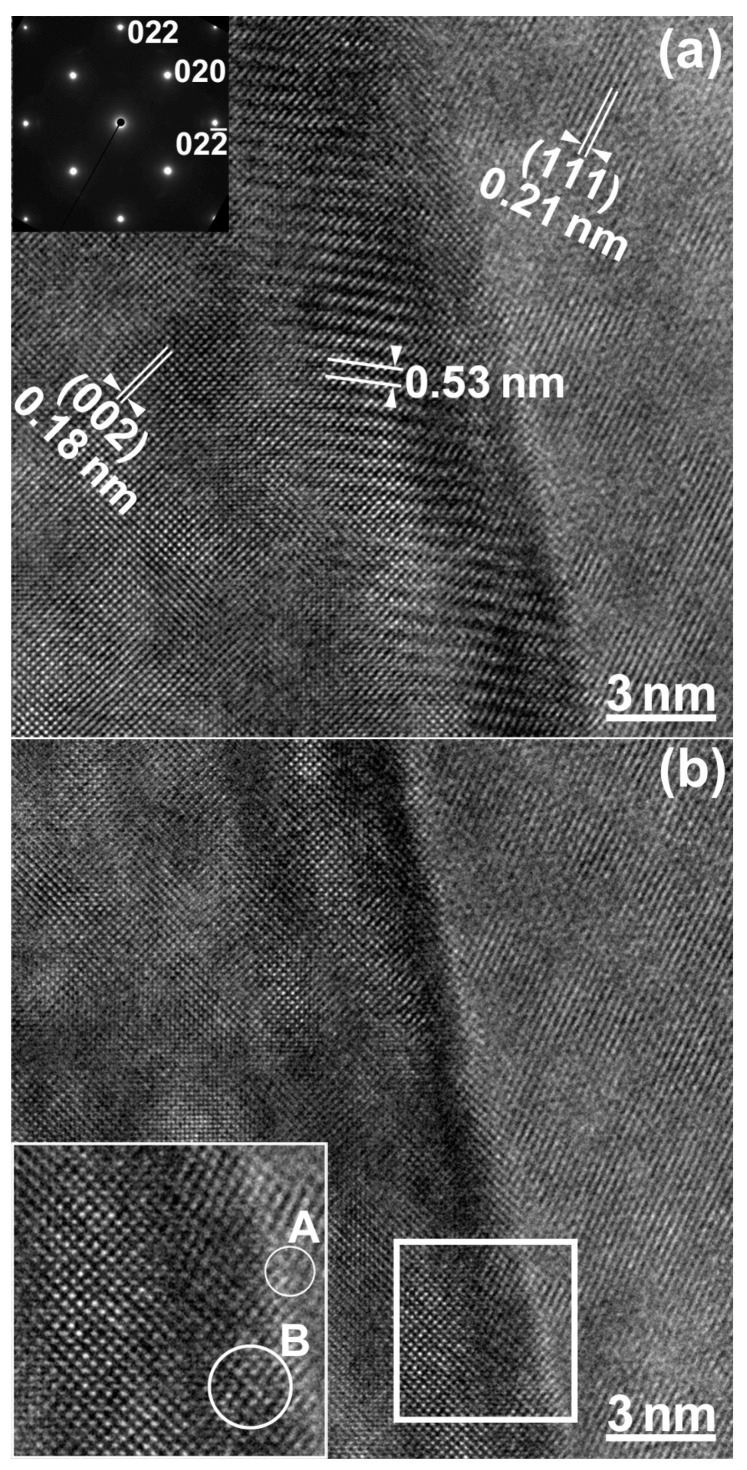
HRTEM images at a lamellar boundary: (**a**) with moiré fringes and (**b**) without moiré fringes.

An HRTEM image of a lamellar boundary between a major layer (left) and a minor layer (right) is shown in Figure 6a. The lamellar structure located on the right side of the boundary showed orthogonal (002) and (020) lattice fringes, while the lamellar structure on the left side of the boundary showed (111) lattice fringes. Moiré fringes with 0.53 nm spacing were observed at the boundary, owing to the overlapping of two adjacent lamellae. These are rotation moiré fringes formed by the interference of the 002 reflection of the major layer and the 111 reflection of the minor layer. An HRTEM image of the boundary without moiré fringes, observed in another field of view of the same interface, is shown in Figure 6b. A magnified image of the area surrounded by the square is shown in the bottom left insert. The circled area A includes an interfacial dislocation, and the circled area B shows lattice distortion. Strain contrast was observed at the boundary, owing to the lattice strain caused by the semi-coherent boundary, as well as solute segregation at the lamellar boundary. This atomic-structure analysis revealed a local interfacial structure that is complementary to the SEM-EBSD results [10,11]. We have confirmed the generality and reproducibility of the above results, obtained by STEM-EDS and HRTEM analysis.

## 4. Conclusions

We have investigated the microstructure and solute segregation of orientation-controlled 316L SS produced by LPBF, using STEM and EDS. The obtained results regarding the *YZ* plane of the material can be summarized as follows:

(1) Cr and Mo were enriched along the lamellar boundaries, as well as in the dislocation cell walls. The segregation of Cr and Mo was ~1 and 1–2 wt %, respectively.
(2) The Cr and Mo contents slightly decreased (by ~0.5 wt %) at the melt-pool boundary.
(3) The solidification cellular microstructures and melt-pool boundaries were visualized with the aid of solute segregation during crystal growth after laser melting.
(4) Mn–Si–O inclusions (10–15 nm in diameter) were distributed along the lamellar boundary, as well as in the dislocation cell wall. It is considered that the grain growth of inclusions can be effectively suppressed by rapid quenching during the LPBF process.
(5) A thin region without cellular microstructures was observed at the melt-pool boundary. The cellular spacing widened near the bottom of the melt pool, owing to the decrease in the cooling rate.
(6) Atomic-structure analysis of the lamellar boundary by HRTEM revealed a local interfacial structure, which is complementary to the SEM-EBSD results.

## Data Availability

The data that support the findings of this study are available within the article.
